# A complexity efficient penta-diagonal quantum smoothing filter for bio-medical signal denoising: a study on ECG

**DOI:** 10.1038/s41598-024-59851-5

**Published:** 2024-05-08

**Authors:** Mostafizur Rahaman Laskar, Sawon Pratiher, Amit Kumar Dutta, Nirmalya Ghosh, Amit Patra

**Affiliations:** 1 G. S. Sanyal School of Telecommunications, Indian Institute of Technology Kharagpur, Kharagpur, 721302 West Bengal India; 2 Department of Electrical Engineering, Indian Institute of Technology Kharagpur, Kharagpur, 721302 West Bengal India

**Keywords:** Computational science, Electrical and electronic engineering, Quantum simulation

## Abstract

Extracting information-bearing signal from a noisy environment has been a practical challenge in both classical and quantum computing formalism, especially in critical signal processing applications. To filter out the effect of noise, we propose a quantum smoothing filter built upon quantum formalism-based circuits applied for electrocardiogram signal denoising. The proposed quantum filter is a conceptually novel framework with an advantage in computational complexity as compared to the existing classical filters, such as discrete wavelet transform and empirical mode decomposition, whereas it achieves similar performance metrics for the accuracy of the filter. Further, we exploit the penta-diagonal Toeplitz structure of the smoothing filter, which gives approximately $48\%$ gate cost reduction for 10 qubit circuit compared to the standard Hamiltonian simulation without structure. The run-time complexity using the quantum matrix inversion technique for the structured matrix is given by $\tilde{{\mathscr {O}}}\left( \frac{\kappa ^2 \text {poly}(\log {N})}{\varepsilon _P}\right)$ for condition number $\kappa$ of the $N\times N$ filter matrix within precision $\varepsilon _P$. Embedding fixed sparsity of the banded matrix, the quantum filter shows potentially better run-time complexity than classical filtering techniques. For the quantifiable research results of our work, we have shown several performance metrics, such as mean-square error and peak signal-to-noise ratio analysis, with a bound of error due to observation noise, simulation error and quantum measurement uncertainty.

## Introduction

Information-bearing signal in practical systems is often corrupted by observation noise, which needs denoising for further analysis^1–3^. In critical biomedical applications such as electrocardiogram (ECG), the signal acquisition process from the human body surface is inevitably contaminated with noise^4^, and is a critical step for ECG signal parameter estimation. Typical noise sources corrupting particular frequency bands of ECG signals are baseline wandering (BW), powerline interference (PLI), i.e., AC interference, electrode motion, and muscle artefacts. The most common additive white Gaussian noise (AWGN) present during channel recording adulterates the entire ECG frequency spectrum^5^. In the context of large-scale ECG analysis, denoised ECG templates are critical for feature extraction^6^, arrhythmia detection^7^, heartbeat classification^8^, and ECG bio-metrics^9,10^.

Statistical signal processing-based ECG denoising techniques like Kalman filtering-based Bayesian frameworks^11^, non-local means (NLM) filtering^5,12,13^, decomposition methods like discrete wavelet transform (DWT)^14^, empirical mode decomposition (EMD)^15^, variational mode decomposition (VMD)^16^, and deep learning (DL)^17^ are routinely used. However, DWT-based ECG denoising discards the low-frequency approximation coefficients completely^16^, EMD and VMD have sample noise sensitivity^16^. The NLM technique is susceptible to the rare-patch effect in the high-frequency QRS-complex^13^, and Kalman filtering require the knowledge of the underlying ECG generating model^11,18^. In addition to massive training data requirements, DL methods are computationally expensive in low-complexity edge computing applications like wearables^6^. The prior art abounds in smoothness prior and quadratic variation (SPQV)-based smoothing filters for many classical signal and image denoising applications. The SPQV technique is often used for modelling non-stationary time series^19^, non-parametric estimation^20^, and surface reconstruction in pattern recognition applications^21^. SPQV-based signal denoising is mainly dependent on the regularization techniques employed in the underlying algorithm, such as penalized least square optimization^22^, Savitzky-Golay filter^23^, Tikhonov regularization^24^, and the band-stop smoothing filter^25^ has recently been applied to ECG denoising.

Although the above-mentioned denoising techniques mitigate the effect of noise, their application in large datasets is a computationally intensive task, especially in critical applications such as continuous ECG monitoring of cardiac patients. The computational run-time complexity of the classical filers largely depends on the underlying matrix inversion methods employed. One of the best classical inversion methods is the conjugate gradient (CG) method with a run-time complexity of ${\mathscr {O}}\left( Nd\sqrt{\kappa }\log {\frac{1}{\varepsilon _P}}\right)$ for *d*-sparse filter matrix ${\textbf{P}}\in {\mathbb {R}}^{N\times N}$. Consequently, there is a need to design a computationally efficient algorithm for signal filtering in the fastest possible way in such critical applications.

This work investigates the possibility of synergy between quantum computation and signal filtering methods to design an efficient quantum filtering algorithm for vital applications like ECG denoising. Quantum computing (QC) and quantum signal processing (QSP) are becoming promising avenues for simulating large-scale problems in science and technology. The recent development of superconducting qubit-based quantum simulators such as the IBM quantum machine provides a practical way to run quantum algorithms (QA) on a quantum computer. QAs have many practical advantages such as efficient computational complexity, fewer physical resources, security and reliability^26^ for processing large data. The recent development in QA such as quantum linear-system solver^27^, quantum principal component analysis^28^, and quantum eigenvalue estimation technique^29^ are based on one or several quantum sub-routines (QSR) including quantum amplitude amplification (QAA), Hamiltonian simulation, quantum Fourier transform (QFT) and quantum phase estimation (QPE)^29–31^.

Given the above background, the contributions of this work are given as follows.

- We propose a quantum smoothing filter (QSF) exploiting the inherent structural property of the filter, which is modelled as a penta-diagonal banded Toeplitz matrix. The structural exploitation is achieved by a proposed Jordan decomposition-based quantum architecture, which requires fewer quantum functional gates compared to the scenario if this structural exploitation would not be considered in the Quantum realm.
- A complexity-efficient quantum filter is designed based on the matrix inversion principle by exploiting the inherent structure of the operator. The modified Hamiltonian-simulation sub-routine is embedded in the matrix inversion process to augment run-time complexity advantage of approximately $\tilde{{\mathscr {O}}}(\frac{c_d \kappa ^2 \log {N}}{\varepsilon _P})$, which is faster than the existing filtering algorithms. Here $c_d$ is a constant, $\kappa$ denotes the condition number of the filter matrix, and $\varepsilon _P$ represents the overall error in the filtering process.
- The efficacy of the proposed quantum framework is measured in terms of mean square error (MSE), which is compared with its classical analogue filtering algorithms. Also, the quantum advantage is compared with the standard Hamiltonian simulation in terms of gate complexity analysis, which measures quantum computational resources. The proposed method’s potency for near-time application is shown in an IBM quantum machine, and the performance is compared with the classical computer. The difference between the classical and the quantum methods due to quantum noise and quantum measurement uncertainty has also been pictured in this work.

### Signal model

Signal filtering is one of the central challenges in signal processing for retrieving information-bearing components, which are embedded with noise, given as:

1 $$\begin{aligned} {{\textbf {y}}}={{\textbf {x}}} + {{\textbf {w}}}, \end{aligned}$$

where ${{\textbf {y}}}\in {\mathbb {C}}^{N\times 1}$ denotes the measured signal, ${\textbf{x}} \in {\mathbb {C}}^{N\times 1}$ represents the desired signal and ${{\textbf {w}}} \in {\mathbb {C}}^{N\times 1}$ is the noise vector. Here our objective is to extract the information-bearing part ${\textbf{x}}$ from the noisy observation ${\textbf{y}}$ as a filtered version, which requires a filtering operation on ${\textbf{y}}$. The proposed quantum framework requires that the signal ${\textbf{y}}$ is encoded in a suitable form for the simulation on a quantum computer. The real computer introduces quantum error to the resultant signal, which is later analyzed in this work.

The signal estimated with the smoothness prior can be written in sampled discrete form as:

2 $$\begin{aligned} {\hat{x}}[i]&= \underset{x[i]}{\arg \min }\ \sum _{j=1}^{N} \left( y[j] - x[j]\right) ^2 + \eta \sum _{j=1}^{N} \left( \nabla ^n x[j] \right) ^2 \end{aligned}$$

where $i=1,\dots , N$, $\eta$ denotes a smoothness trade-off parameter, and $\nabla ^n$ is the *n* *th*-order difference approximation of the derivative given by

3 $$\begin{aligned} \nabla ^n x[i] = \sum _{j=0}^{n} (-1)^j ~{} ^nC_{j} x[i\pm j], \end{aligned}$$

with $^nC_{j}$ as the binomial coefficient. The solution of (2) becomes

4 $$\begin{aligned} \hat{{\textbf{x}}}&= \left( I+ \eta {\textbf{D}}^T{\textbf{D}} \right) ^{-1} {\textbf{y}}\nonumber \\&= {\textbf{P}}^{-1} {\textbf{y}} ~(\text {assuming}~ {\textbf{P}}=I+ \eta {\textbf{D}}^T{\textbf{D}} ), \end{aligned}$$

where the matrix ${\textbf{I}}$ denotes the identity matrix of order N and ${\textbf{D}} \in {\mathbb {R}}^{N-2 \times N}$ is a banded Toeplitz matrix obtained from the backward difference operator with a band ($d_0, \dots , d_p$) of the form given as

5 $$\begin{aligned} {\textbf{D}}= \begin{bmatrix} d_0 &{}d_1 &{}d_2&{}\dots &{} d_p &{}0 &{}\dots &{}0\\ 0 &{} d_0 &{} d_1 &{} \dots &{}d_{p-1}&{} d_p&{} \ddots &{}\vdots \\ \vdots &{} \ddots &{} \ddots &{}\ddots &{} \ddots &{}\ddots &{}\ddots &{} 0 \\ 0 &{}\dots &{} 0 &{} d_0 &{}d_1 &{}d_2&{}\dots &{} d_p\\ \end{bmatrix}. \end{aligned}$$

For *N*-samples, and 2*nd* order smoothness prior the matrix ${\textbf{D}}$ has the following form

6 $$\begin{aligned} {\textbf{D}}= \begin{bmatrix} 1 &{}-2 &{}1 &{}0 &{}\dots &{}0\\ 0 &{} 1 &{} -2 &{} 1 &{} \ddots &{}\vdots \\ \vdots &{} \ddots &{} \ddots &{}\ddots &{} \ddots &{} 0 \\ 0 &{}\dots &{} 0 &{} 1 &{} -2 &{} 1\\ \end{bmatrix}. \end{aligned}$$

Note that the elements of the band can be taken from a suitable Kernel function. We have a kernel of $[1,~-2,~1]$ for the ECG signal denoising with the second-order smoothing. The transfer function for the filter in our consideration is a low-pass smoothing filter (LPSF), expressed in ${\textbf{Z}}$-domain with angular frequency $\omega$ given by

7 $$\begin{aligned} {\textbf{H}}_{LP}(z)&= \frac{1}{1+ \eta (1-z^{-1})^n (1-z)^n},\nonumber \\ {\textbf{H}}_{LP}(e^{j\omega })&= \frac{1}{1 +\eta (2 \sin \frac{\omega }{2})^{2n}}, \end{aligned}$$

where *n* is the order of the derivative to get the operator ${\textbf{D}}$. In our approach, we have kept this simplest filter configuration due to the banded symmetric structure of the filter operator ${\textbf{P}}$. One can further improve the filter response by using the high-pass and band-stop filter^25^ at the cost of increasing complexity. The value of the hyper-parameter $\eta$ can be chosen based on the filter order and cut-off frequency of the Fourier frequency response ${\textbf{H}}_{LP}(e^{j\omega })$.

**Figure 1:**
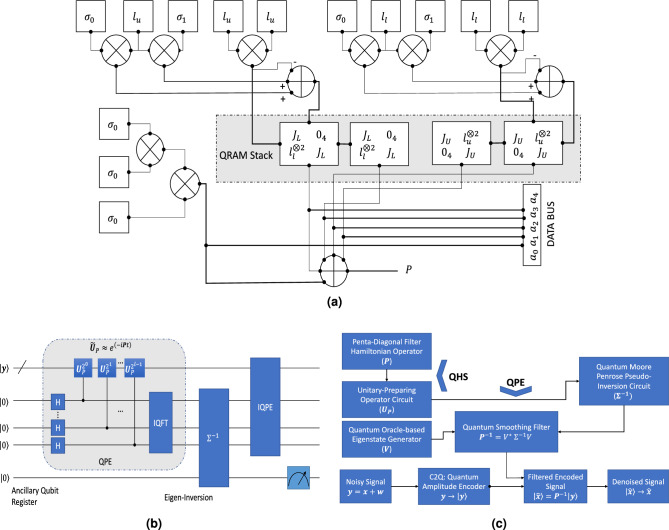
Quantum architecture and flow-diagram for signal denoising. (**a**) The proposed quantum architecture for a penta-diagonal banded Toeplitz Hamiltonian of size 8×8 using elementary quantum gates: Here, dark dots represent connections, the circle ⊗ notation represents tensor operation, circle ⊕ represents the adder circuit, σ0,σ1, lu, ll , JU, JL are identity gate, Pauli-x operator, upper-ladder, and lower-ladder operator, upper-Jordan gate, and lower-Jordan gates respectively, a0, . . . ,a4 are the filter coefficients with a1 = a3, a2 = a4, and QRAM represents quantum random access memory. (**b**) A quantum circuit of a quantum filter using the quantum phase estimation circuit using the proposed quantum filter: the sub-system in as shown in (**a**) is used here as controlled unitary $\tilde{{\textbf{U}}}_P$. (**c**) Flow diagram of signal denoising using the proposed quantum filter: Here, C2Q denotes the classical to quantum encoding block, the sub-routine shown in (**b**) is used here as QPE sub-system.

### Quantum formalism

#### Signal encoding

Among several quantum encoding methods, such as basis encoding, amplitude encoding, and Hamiltonian encoding, we consider the amplitude encoding technique for preparing the quantum states efficiently with $\log _2 (N)$ qubits for encoding *N*-length observation vector. The ECG signal vector can be amplitude encoded in qubits as

8 $$\begin{aligned} {|{{{\textbf {x}}}}\rangle } =\sum _{i=1}^{l} {\alpha }_i {|{x}\rangle }_i ~\text {for}~ i\in \{1,\dots ., l\}, \end{aligned}$$

where the probability amplitude for the $i^{th}$-basis vector ${|{x}\rangle }_i$ is given by ${\alpha }_i= \frac{{x}_i}{\Vert {{\textbf {x}}} \Vert } ~\text {for}~ x_i \in {\textbf{x}}, ~\text {and}~ i\in \{1,\dots ., l\}$ with a *l*-basis set. The amplitude-encoded quantum state vector corresponding to the observation ${\textbf{y}}$ can be prepared similarly as

9 $$\begin{aligned} {|{{{\textbf {y}}}}\rangle } = \sum _{i=1}^{l} {\beta }_i {|{y}\rangle }_i, \end{aligned}$$

with probability amplitude of $\beta _i$ for the *i* *th* basis vector ${|{y}\rangle }_i$. We are interested in getting an estimate of the pure ECG signal, i.e., $\hat{{\textbf{x}}}$ from the noisy encoded observation vector ${|{{\textbf{y}}}\rangle }$.

#### Proposed Hamiltonian simulation exploiting penta-diagonal matrix operator

A quantum evolution operator is required to perform a quantum formalism-based SPQV filtering approach. Here, we propose a quantum algorithm with an efficient quantum architecture for the filter operator ${\textbf{P}}$. The filtering operation can be performed using a quantum evolution operator (a unitary matrix here) based on the matrix ${\textbf{P}}$. We embed quantum Hamiltonian simulation (QHS) to get the unitary operator as ${\textbf{U}}_P=\exp (-i{\textbf{P}}t)$, where the evolution time is *t*. In reality, the Hamiltonian simulation is performed with an approximation for optimal usage of quantum resources. Standard QHS methods simulate the Hamiltonian simulation with several techniques such as product formula^32^, truncated Taylor series^33^, qubitization^34^, quantum walk^35^, and Quantum signal processing algorithm^36^. The approximated unitary $\tilde{{\textbf{U}}}_P$ prepared for the operator ${\textbf{P}}$ incurs an error given by $\Vert \tilde{\textbf{U}}_P - {\textbf{U}}_P \Vert \le \varepsilon _P$.

In standard QHS approaches such as Trotterization and Taylor series method, the Hamiltonian is often presented on Pauli-basis. In general, it requires that the Hamiltonian is a symmetric matrix, and the advantage of computational complexity is often dependent on the sparsity of the underlying Hamiltonian in the QHS. In this work, considering the kernel as $[1~-2~1]$, we get the Hamiltonian operator ${\textbf{P}}$ given by (10).

Here, the coefficients $a_0, a_1$, and $a_2$ are generated from the relationship ${\textbf{P}}=I+ \eta {\textbf{D}}^T{\textbf{D}}$. The matrix ${\textbf{P}}$ is a banded and penta-diagonal matrix with $5N-6$ non-zero elements. Hence, it can be considered a sparse Hamiltonian, and the quantum complexity advantage can be significant with large dimension *N*. The matrix ${\textbf{P}}$ is symmetric, so it can be decomposed with the basis obtained from Pauli operators.

10 $$\begin{aligned} {\textbf{P}}= \begin{bmatrix} a_0 &{}a_1 &{}a_2 &{}0 &{} 0 &{}0 &{} \dots &{}0\\ a_1 &{}a_0 &{}a_1 &{}a_2 &{} 0 &{}0 &{} \ddots &{}\vdots \\ a_2 &{}a_1 &{}a_0 &{}a_1 &{} a_2 &{}0 &{} 0 &{}0\\ 0 &{}a_2 &{}a_1 &{} a_0 &{}a_1 &{} a_2 &{}0 &{}0\\ \vdots &{}\ddots &{}\ddots &{}\ddots &{}\ddots &{} \ddots &{}\ddots &{} \vdots \\ 0 &{}0 &{}0 &{}a_2 &{} a_1 &{}a_0 &{} a_1 &{}a_2\\ \vdots &{}\ddots &{}0 &{}0 &{} a_2 &{}a_1 &{} a_0 &{}a_1\\ 0 &{}\dots &{}0 &{}0 &{} 0 &{}a_2 &{} a_1 &{}a_0\\ \end{bmatrix} \in {\mathbb {R}}^{N\times N}. \end{aligned}$$

To exploit the Toeplitz structure embedded in the filter matrix, we choose the Jordan-normal form as the basis of decomposition. The 1-sparse $N\times N$ Jordan matrix gives the super-diagonal basis, which is given as

11 $$\begin{aligned} {\textbf{J}}_N=\begin{bmatrix} 0 &{} 1&{} 0 &{} \dots &{} 0\\ 0 &{} 0 &{} 1 &{}\ddots &{} \vdots \\ 0 &{} \ddots &{}\ddots &{} \ddots &{} 0\\ \vdots &{} \ddots &{} \ddots &{}\ddots &{} 1\\ 0 &{} \dots &{} 0 &{} 0 &{}0 \end{bmatrix}. \end{aligned}$$

The ${\textbf{J}}_N^{\dagger }$ is its symmetric form and provides the off-diagonal basis below the diagonal. The quantum-architecture realization of the Jordan form for $N=8$ is given in Fig.1a.

##### Lemma 1

The Hamiltonian matrix ${\textbf{P}}$ can be decomposed in Jordan-basis as a combination of 4 off-diagonals (using elementary quantum gates) and 1-diagonal basis (with tensor product of identity gates as a basis) scaled by the filtered coefficient as follows

12 $$\begin{aligned} {\textbf{P}}=a_0{\textbf{I}}_N + \sum _{i=1}^{2}\times a_i {\textbf{J}}_N^i + \sum _{i=1}^{2}\times a_i {\textbf{J}}_N^{\dagger i}. \end{aligned}$$

13 $$\begin{aligned} {\textbf{P}}=a_0 {\textbf{I}} + a_1 \left( \begin{bmatrix} 0 &{} 1&{} 0 &{} \dots &{} 0\\ 0 &{} 0 &{} 1 &{}\ddots &{} \vdots \\ 0 &{} \ddots &{}\ddots &{} \ddots &{} 0\\ \vdots &{} \ddots &{} \ddots &{}\ddots &{} 1\\ 0 &{} \dots &{} 0 &{} 0 &{}0 \end{bmatrix} + \begin{bmatrix} 0 &{} 0&{} 0 &{} \dots &{} 0\\ 1 &{} 0 &{} 0 &{}\ddots &{} \vdots \\ 0 &{} \ddots &{}\ddots &{} \ddots &{} 0\\ \vdots &{} \ddots &{} \ddots &{}\ddots &{} 0\\ 0 &{} \dots &{} 0 &{} 1 &{}0 \end{bmatrix}\right) + a_2 \left( \begin{bmatrix} 0 &{} 0&{} 1 &{} \dots &{} 0\\ 0 &{} 0 &{} 0 &{}\ddots &{} \vdots \\ 0 &{} \ddots &{}\ddots &{} \ddots &{} 1\\ \vdots &{} \ddots &{} \ddots &{}\ddots &{} 0\\ 0 &{} \dots &{} 0 &{} 0 &{}0 \end{bmatrix} + \begin{bmatrix} 0 &{} 0&{} 0 &{} \dots &{} 0\\ 0 &{} 0 &{} 0 &{}\ddots &{} \vdots \\ 1 &{} \ddots &{}\ddots &{} \ddots &{} 0\\ \vdots &{} \ddots &{} \ddots &{}\ddots &{} 0\\ 0 &{} \dots &{} 1 &{} 0 &{}0 \end{bmatrix}\right) . \end{aligned}$$

##### Proof

The Hamiltonian ${\textbf{P}}$ is defined as ${\textbf{I}}+\eta {\textbf{D}}^{T}{\textbf{D}}$. For a kernel $[1~-2~1]$, the matrix ${\textbf{D}}$ is tri-diagonal and ${\textbf{D}}^T{\textbf{D}}$ is penta-diagonal. As a consequence, ${\textbf{P}}= {\textbf{I}}+\eta {\textbf{D}}^{T}{\textbf{D}}$ is penta-diagonal as well as Toeplitz as shown in (10). The diagonal of ${\textbf{P}}$ can be implemented with tensor products of identity gates scaled by the coefficient $a_0$. The effective matrix can be decomposed into the sum of five sparse matrices, including $a_0 {\textbf{I}}$, corresponding to the diagonal matrix. As ${\textbf{P}}$ is Toeplitz, each off-diagonal can be represented with $\tilde{{\mathscr {O}}}(1)$ sparse matrices (${\textbf{J}}_N$ or ${\textbf{J}}_N^{\dagger }$ and their square) scaled by corresponding coefficients $a_1, a_2$ as shown in (13). $\square$

***An Example of the Proposed Architecture :*** We show an example architecture of ${\textbf{P}}$ of size $8\times 8$ based on the lemma with the quantum gate complexity reduction by augmenting the filter operator’s structural advantage as shown in Fig. 1a. Elementary gates such as Pauli and Hadamard gates are available in real quantum machines such as IBM-QISKIT^37^. In Fig. 1a, the ladder gates are used, which can be implemented using combinations of Pauli gates as follows

14 $$\begin{aligned} l_u&= \frac{1}{2}(\sigma _1 + i\sigma _2) = \begin{bmatrix} 0 &{} 0\\ 1 &{} 0 \end{bmatrix},~\text {and} \end{aligned}$$

15 $$\begin{aligned} l_l&= \frac{1}{2}(\sigma _1 - i\sigma _2) = \begin{bmatrix} 0 &{} 1\\ 0 &{} 0 \end{bmatrix}, \end{aligned}$$

where $\sigma _1$ and $\sigma _2$ are Pauli-*x* and Pauli-*y* gates respectively. In this circuit, we have used Jordan gates ${\textbf{J}}_L$, and ${\textbf{J}}_U$ which can be obtained as follows

16 $$\begin{aligned} {{\textbf {J}}}_L&= \left( \varvec{\sigma }_0 \otimes l_l \right) + \left( l_l \otimes \varvec{\sigma }_x \right) - \left( l_l \otimes l_l \right) \nonumber \\&=\begin{bmatrix} 0&{}1&{}0&{}0\\ 0&{}0&{}1&{}0\\ 0&{}0&{}0&{}1\\ 0&{}0&{}0&{}0 \end{bmatrix}, ~\text {and}\end{aligned}$$

17 $$\begin{aligned} {{\textbf {J}}}_U&= \left( \varvec{\sigma }_0 \otimes l_u \right) + \left( l_u \otimes \varvec{\sigma }_x \right) - \left( l_u \otimes l_u \right) \nonumber \\&=\begin{bmatrix} 0&{}0&{}0&{}0\\ 1&{}0&{}0&{}0\\ 0&{}1&{}0&{}0\\ 0&{}0&{}1&{}0 \end{bmatrix}. \end{aligned}$$

In the circuit diagram Fig. 1a, $\sigma _0$ is the $2\times 2$ identity gate, and ${\textbf{0}}_4$ denotes the $4\times 4$ zero-matrix.

***Determination of approximate unitary*** $\tilde{{\textbf{U}}}_P$: The implementation of the ideal unitary operator ${\textbf{U}}_P$ is expensive. Hence, an approximate unitary operator corresponding to ${\textbf{P}}$ can be prepared practically via the quantum Hamiltonian simulation (QHS) within an error of $\varepsilon _P$ as follows

18 $$\begin{aligned} \Vert \tilde{{\textbf{U}}}_P - \exp \left( {-i{\textbf{P}} t}\right) \Vert \le \varepsilon _P. \end{aligned}$$

The problem in (18) can be addressed with standard QHS approaches such as Trotter-Suzuki approximation^38,39^, Quantum walk^40^, and Taylor series approximation^33^. Here, we proceed with the Taylor series truncation method for the approximation of the unitary matrix $\tilde{{\textbf{U}}}_P$ up-to-order *L* as

19 $$\begin{aligned} \tilde{{\textbf{U}}}_P&= \sum _{l=0}^{L-1}\frac{\left( -i{\textbf{P}} t\right) ^l}{l!} + \varepsilon _P. \end{aligned}$$

#### Quantum matrix inversion

We employ the HHL matrix inversion method^27^ in which the observation vector ${|{{\textbf{y}}}\rangle }$ needs to be decomposed in the eigenbasis of ${\textbf{P}}$ as $|{\mathbf{y}}\rangle = \sum {_{{j = 1}}^{N} } \beta _{j} |{\mathbf{u}}\rangle _{i}$ via the QPE approach^27,41^. The QPE sub-routine is employed to get the eigenvalues of the operator ${\textbf{P}}$. The approximated unitary matrix $\tilde{{\textbf{U}}}_P$ is applied as controlled *U*-gate in the QPE circuit as shown in Fig. 1b, which impacts the phases of the ${|{{\textbf{1}}}\rangle }$. The QPE circuit estimates the phases $\theta _j\in \left[ 0,1\right)$ such that $\tilde{{\textbf{U}}}_P{|{{\textbf{u}}}\rangle }_j=\exp {(2i\pi \theta _j)}{|{{\textbf{u}}}\rangle }_j$ for $j\in {1,\dots , N}$. Applying the Fourier transform sub-routine on the first register (and converting Fourier basis ${|{k}\rangle }$ to the eigenbasis ${|{{\tilde{\lambda }}_k}\rangle }$), we obtain the state

20 $$\begin{aligned} {|{\varphi }\rangle }_1= \sum _{j=1}^{N} \sum _{k=0}^{T-1} \delta _{k|j} \beta _j {|{{\tilde{\lambda }}_k}\rangle } {|{{\textbf{u}}}\rangle }_j, \end{aligned}$$

where $\delta _{k|j}$ is a normalizing factor, ${\tilde{\lambda }}_k=\frac{2 \pi k}{t_0}$ with $t_0 ={\mathscr {O}}(\frac{\kappa }{\varepsilon _P})$, and *T* can be chosen sufficiently large for the conditional evolution $\sum _{\zeta =0}^{T-1}{|{\zeta }\rangle }{\langle {\zeta }|} \otimes \exp {(i{\textbf{P}}t)}$ with $t:=\frac{\zeta t_0}{T}$. Considering $\delta _{k|j}=1$ in ${|{\varphi }\rangle }_1$, ${\tilde{\lambda }}_k={\hat{\lambda }}_k$ and applying conditional rotation yields

21 $$\begin{aligned} {|{\varphi }\rangle }_2= \sum _{j=1}^{N} \sum _{k=0}^{T-1} \beta _j {|{{\textbf{u}}_j}\rangle } \left( \sqrt{1-\frac{C_m^2}{{\hat{\lambda }}_k}}{|{{\textbf{0}}}\rangle } +\frac{C_m}{{\hat{\lambda }}_k}{|{{\textbf{1}}}\rangle }\right) , \end{aligned}$$

where $C_m$ can be chosen as ${\textbf{O}}(\frac{1}{\kappa })$. The filtered signal is retrieved after multiple measurements of ${|{\varphi }\rangle }_2$ in state ${|{{\textbf{1}}}\rangle }$ given by

22 $$\begin{aligned} {|{{\textbf{x}}}\rangle }= \sum _{j=1}^{N} \beta _j {\hat{\lambda }}_j^{-1} {|{{\textbf{u}}_j}\rangle }= {\textbf{P}}^{-1}{|{{\textbf{y}}}\rangle }. \end{aligned}$$

### Proposed algorithm

We have shown the pseudo-code for signal denoising using the proposed quantum formalism in Algorithm 1.

**Algorithm 1:**
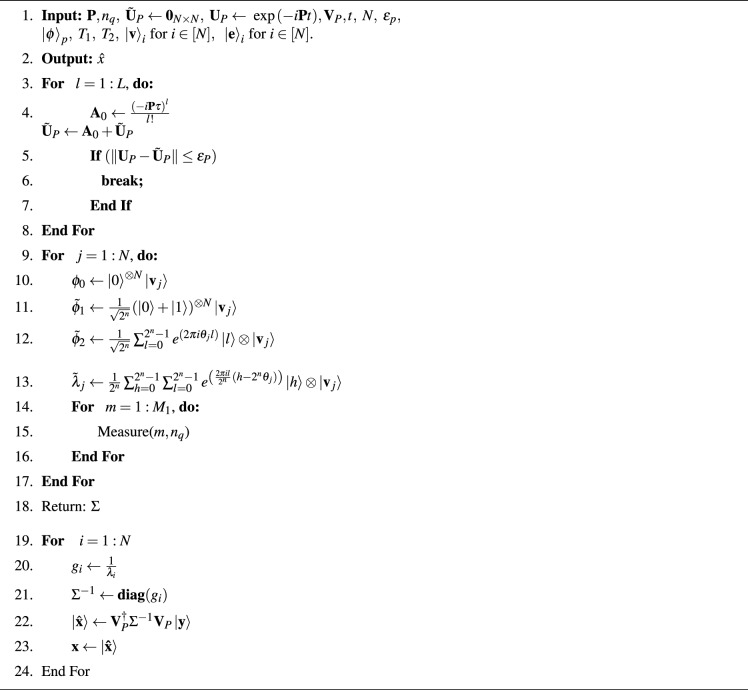
Proposed QSF for signal denoising.

In Fig. 1c, a flow diagram of the quantum signal denoising process is shown. The pseudo-code of the proposed quantum filtering method, as shown in Algorithm 1, is summarized as follows.

- Noisy (ECG) signal is encoded with quantum amplitude encoding as ${|{{\textbf{x}}}\rangle }$ using qubit size of $n_q$.
- The symmetric filter operator is processed to prepare an approximate unitary operator using quantum Hamiltonian simulation (QHS). We have used the Taylor-series truncation method to approximate the unitary matrix $\tilde{\textbf{U}}$ with approximation error $\varepsilon _p$.
- Given a preparation of the eigenstate vectors in the oracle as ${|{{\textbf{v}}_j}\rangle }$, the quantum phase estimation (QPE) circuit estimates the eigenvalues of the filter matrix ${\textbf{P}}$ following superposition of the input qubits, application of control rotation gates, and the inverse quantum Fourier transform (IQFT) given by
  23 $$\begin{aligned} {\tilde{\lambda }}_j \leftarrow \frac{1}{2^{j}} \sum _{h =0}^{2^j-1} \sum _{l=0}^{2^j -1} e^{\left( \frac{2\pi i l}{2^j} \left( h-2^j\theta _{j}\right) \right) }{|{h}\rangle }\otimes {|{{\textbf{v}}_j}\rangle }. \end{aligned}$$
- Following the quantum eigen-inversion method, the Moore-Penrose pseudo-inverse of the filter matrix is prepared as
  24 $$\begin{aligned} {\textbf{P}}^{-1}= {\textbf{V}}_P^{\dagger } \Sigma ^{-1} {\textbf{V}}_P, \end{aligned}$$
  where $$\Sigma ^{-1}=\textbf{diag}\left( \frac{1}{{\hat{\lambda }}_1}, \dots , \frac{1}{{\hat{\lambda }}_N} \right)$$.
- The encoded filtered signal is obtained as follows ${|{{\textbf{x}}}\rangle }={\textbf{P}}^{-1}{|{{\textbf{y}}}\rangle }$. The filtered signal from the encoded form to the discrete signal can be obtained by multiplying it with a suitable factor.

In Fig. 1a, we show a quantum circuit of the proposed quantum filter. The QPE circuit produces the eigenvalues of the operator ${\textbf{P}}$ whose precision depends on the length of the input qubit size and the Hamiltonian approximation algorithm. The eigen-inversion and inverse-QPE circuit generates the filter operator ${\textbf{P}}^{-1}$. The Hamiltonian simulation for the matrix ${\textbf{P}}$ can be performed using the Pauli bases (e.g., $\sigma _x$, $\sigma _y$, $\sigma _z$), as it is in Hermitian matrix form. To exploit the structural benefits of the banded-Toeplitz matrix by sparse decomposition, we propose the Jordan gate-based Hamiltonian simulation for the matrix inversion problem.

#### Lemma 2

A Hamiltonian operator, ${\textbf{P}} \in {\mathscr {C}}^{N\times N}$ of the form penta-diagonal banded-Toeplitz can be realized using elementary quantum gates with computational resources (as a function of input qubit size $n_q$) is given by

25 $$\begin{aligned} C_g = n_q2^{n_q}. \end{aligned}$$

#### Proof

The computational gate counts (which is a measure of computational resources in Noisy Intermediate Scale Quantum (NISQ) for designing the Hamiltonian, ${\textbf{P}} \in {\mathbb {R}}^{N\times N}$ as a function of input qubit size ($n_q$) is discussed as follows:

- We need $n_q=\log _2N$ number of Identity gates ($\sigma _0$) for preparing the principal diagonal.
- We have 4 off-diagonals in the filter Hamiltonian matrix, ${\textbf{P}}$, which can be prepared with combinations of Jordan gates. Using the recursive implementation of the Jordan gates, we need two Jordan sub-circuits ${\textbf{J}}_{\frac{N}{2}}$ and an additional upper ladder operator. We require $[\log _2 {\frac{N}{2}}]$ elementary $l_u$ gates to implement a ladder operator $l_u{}_{\frac{N}{2}}$.
- A $4\times 4$ Jordan gate ${\textbf{J}}_4$ requires four elementary gates (One $\mathbf {\sigma }_{0}$, two lower ladder gates ($l_L$ for ${\textbf{J}}_4$, and $l_u$ for ${\textbf{J}}_4^{\dagger }$), and one $\mathbf {\sigma }_{x}$, respectively). Using the recursive architecture of the Jordan block, the elementary gates required for the implementation of the banded Toeplitz matrix are given by $\varvec{\Theta }(N\log N)$. Hence, the computational gate complexity, $C_g$ in terms of input qubit size, is given by $\varvec{\Theta }(n_q2^{n_q})$.

$$\square$$

***Note***: Given the length of the ECG signal, *N*, we choose $n_q=\lceil {\log N}\rceil$ for efficient quantum encoding of the ECG signal. The basic operations needed for a unitary matrix simulation (${\textbf{U}}(2^{n_q})$) is given by $\varvec{\Theta }({n_q}^3 4^{n_q})$ (Section VIII in^42^). Here, we see that considering each gate to perform a basic operational unit, the banded Toeplitz-patterned matrix needs an overall lesser number of quantum gates, as shown in Table 1.

**Table 1:** Calculation of gate counts.

Hamiltonian simulation	Gate counts
Standard unitary simulation	$\varvec{\Theta }({n_q}^3 4^{n_q})$
Banded Toeplitz structured unitary simulation	$\varvec{\Theta }(n_q2^{n_q})$

## Results

This section discusses the experimental evaluation of our proposed quantum filter on synthetic and real-world noisy ECG signals. The synthetic ECG records are generated using ECGSYN—A realistic ECG waveform generator (https://physionet.org/content/ecgsyn/1.0.0/). The real-world ECG data are taken from the MIT-BIH Arrhythmia database^43^, where the ECG signals are sampled at 360 Hz with 11-bit resolution. A comparative evaluation with the existing ECG denoising methods like EMD^15^, NLM^12,13^, and DWT^14^ is carried out to show the effectiveness of the proposed method. The uncertainty factors considered in the overall quantum algorithm are observation noise, quantum simulation error, and quantum measurement uncertainty. The experiments are partly simulated on a classical computer with MATLAB and partly on an IBM ’Statevector’ quantum simulator. The choice of simulation parameters and their values are given in Table 2.

**Table 2:** Simulation parameters.

Parameters	Numerical value
Simulation time (*t*)	0.2 second
Precision (${\varepsilon }_P)$	0.01
$\Vert {\textbf{P}} \Vert _2$	1
Dimension of noisy signal ${\textbf{y}}$ (*N*)	600, 2351
Filter coefficient ($\eta$)	*N*/25
Matrix dimension of ${\textbf{P}}$	$N\times N$
Qubit size	$\lceil {2\log _2{N}}\rceil + l$
SNR (in dB)	5 to 25 dB.
Kernel	$[1, -2, 1]$

**Figure 2:**
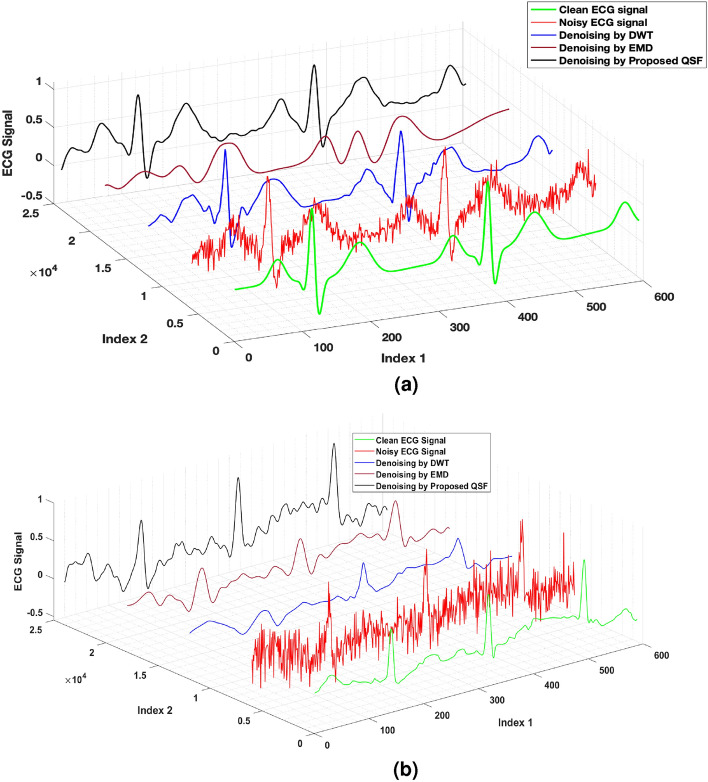
Performance of the proposed QSF for healthy and diseased datasets in comparison with classical DWT^1^, and EMD^44^ methods. (**a**) A Snapshot of the clean ECG signal of a healthy dataset and the denoised signal using different methods. (**b**) A Snapshot of the clean ECG signal from a diseased (atrial fibrillation) dataset and the denoised signal using different methods.

**Figure 3:**
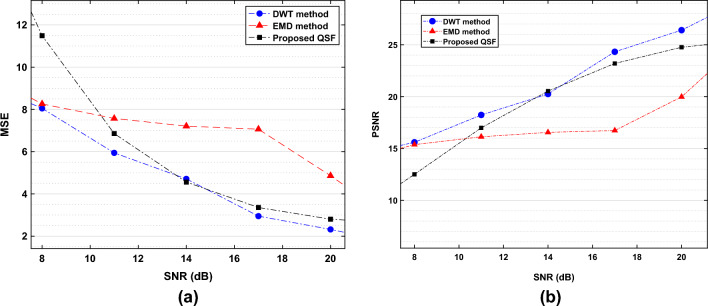
Performance of the proposed QSF in comparison with classical DWT^1^ and EMD^44^. (**a**) MSE performance of the QSF. (**b**) PSNR performance of the QSF.

**Figure 4:**
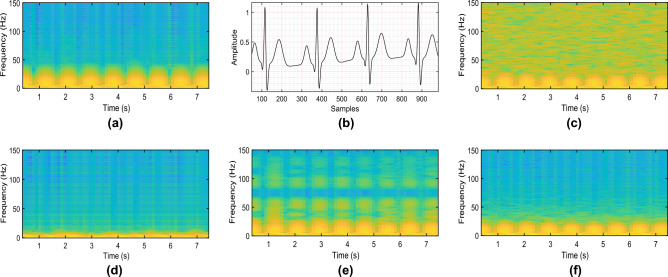
Time-amplitude waveform and time-frequency spectrogram of clean ECG, noisy signal and denoised signals. (**a**) Spectrogram of clean ECG. (**b**) Waveform of clean ECG. (**c**) Spectrogram of noisy ECG (σw = 0.05). (**d**) Spectrogram with EMD. (**e**) Spectrogram with DWT. (**f**) Spectrogram with QSF.

### Accuracy of the filter in comparison to classical methods

Figure 2 shows the performance of the proposed QSF approach applied to denoise the ECG signal. AWGN noise with a varying signal-to-noise ratio (SNR) from 5 to 25 dB is added to the clean ECG signal for evaluating the performance of the proposed algorithm. A snap of the denoised signal from the filter response is shown in Fig. 2a. The method is compared with two widely used classical algorithms viz., discrete wavelet transform (DWT)^1^ and the empirical mode decomposition (EMD)^44^. The proposed QSF attains the denoising performance closer to the classical DWT method, as shown in the black-coloured ECG curve. As the Moore-Penrose pseudo-inverse of the filter matrix has an inbuilt regularization with the parameter $\lambda$, it regularizes perturbation up to a certain level. As a consequence, it gives prominent filtering performance as compared to the EDM technique. In Fig. 2a, the index 1 shows time samples, the signal amplitude of the ECG is shown as the vertical axis, and index 2 is drawn to separate the filter responses of different algorithms. Further, to evaluate the performance of the proposed QSF, we take a diseased dataset (with atrial fibrillation) in the presence of AWGN noise. A snapshot of the filtering performance of different classical filters and the proposed quantum filter at SNR of 15 dB is shown in Fig. 2b. The peaks are perfectly detected by the proposed QSF filter at regularization factor $\lambda =\frac{N}{15}$.

Further, The performance is evaluated with mean square error (MSE) and the peak signal-to-noise ratio (PSNR) metrics, defined for $M_1$ number of samples as follows

26 $$\begin{aligned} \text {MSE}&:= \frac{1}{M_1}\sum _{j=1}^{M_1}\Vert \hat{{\textbf{x}}}(j) - {\textbf{x}}(j)\Vert _2, \end{aligned}$$

27 $$\begin{aligned} \text {PSNR}&:= 20~\log _{10} ({\text {MAX}_{x}}) - 10~\log _{10} ({\text {MSE}}), \end{aligned}$$

where $\text {MAX}_{x}$ denotes the maximum possible signal amplitude in the ECG signal ${\textbf{x}}$. Lower MSE and higher PSNR signify the better quality of signal reconstruction of a filter. In Fig. 3a and b, we have shown the MSE and PSNR performance of the proposed QSF algorithm for varying noise levels. At an SNR of 10 dB, the quantum filter has an improved MSE performance of approximately $43.71\%$ in comparison to the classical EMD method, which is close to the classical DWT algorithm (it has improved MSE performance of $41.78\%$ approximately as compared to EMD). From 14 dB SNR and above, the DWT algorithm outperforms the denoising performance where the proposed QSF follows similar performance characteristics closer to the DWT.

Similar results are also reported in PSNR values. As shown in Fig. 3b, the PSNR curve improves with increasing SNR values. In comparison with the EMD method, both the classical DWT and the proposed quantum filter have significant performance improvement. At an SNR of 17 dB, the proposed QSF has an improved PSNR of $25.24\%$ approximately (DWT has an improved PSNR of $26.08\%$ approximately) in comparison with the classical EMD method.

***Note***: As presently, quantum gates and qubits are not ideal and possess inherent noise sources (which is considered in the simulation), the proposed quantum filter has a slightly degraded performance in the lower SNR levels, which is expected to be eliminated in the near future with error-tolerant quantum hardware. Here, the proposed QSV filter shows performance equivalent to that of classical filters at moderate to high SNR values. Here, our motivation in this work is to design a quantum formalism-based filter i.e., QSF, which can provide quantum speed-up for faster data processing on a quantum computer with excellent run-time complexity without compromising the filter’s accuracy.

The spectrogram plot of the ECG signal is shown in Fig. 4. The time-frequency domain plot of ECG signals shows the high visual quality and accuracy of the filtering methods in reconstructing the estimated ECG. The spectrogram of the clean ECG is shown in Fig. 4a corresponding to the waveform in the time-amplitude response given in Fig. 4b. AWGN is added to the clean ECG signal with a standard deviation of 0.05, which has a spectrogram shown in Fig. 4c. Signal denoising response with the EMD method (Fig. 4d) shows substantially degraded signal reconstruction. The proposed quantum smoothing filter performs excellent signal denoising, demonstrated in Fig. 4f, which matches the energy profile closer to the classical DWT approach given by Fig. 4e.

**Figure 5:**
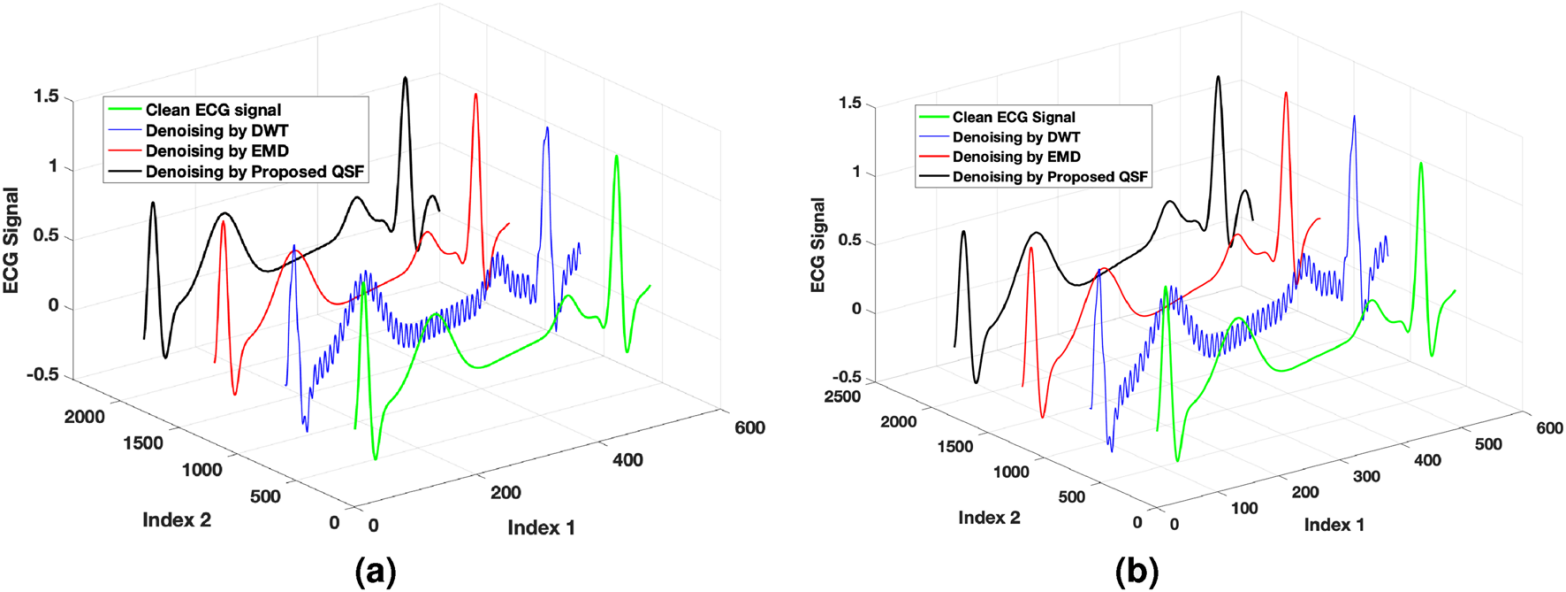
A snapshot of the clean ECG signal and the denoised version using different methods for different noise types. (**a**) Corrupted with AC interference. (**b**) Corrupted with AC interference and baseline wandering.

### Proposed QSF’s performance to other ECG noises: a case study

Figure 5 shows the performance comparison of the proposed QSF method to denoise the ECG signal corrupted with AC interference and BW. With the AC noise and BW variation, the performance of the classical DWT method is degraded, whereas the proposed QSF method shows filtering performance similar to the EMD algorithm. Here, we have chosen the regularization parameter in the order of data dimensionality for its optimal performance. In both the AC interference and BW cases, the results in Fig. 5 show the efficacy of the proposed quantum formalism in ECG signal denoising.

**Figure 6:**
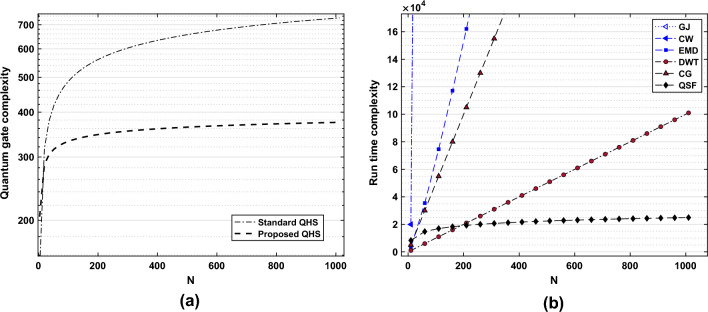
Comparison of computational complexity. (**a**) Quantum gate complexity. (**b**) Run time complexity

### Comparison of computational complexity

The primary motivation behind designing a quantum filter is to get computational advantage while processing large ECG datasets. Here, we demonstrate two key aspects, viz., the quantum gate complexity (resources in terms of elementary quantum gate operations) and the run-time complexity in terms of time operations. The run-time complexity is often resource-independent, and hence, it may be used to compare the complexity between a classical filter and a quantum filter. On the other side, the quantum gate complexity is demonstrated to show the further improvement of the quantum Hamiltonian simulation, which is a critical sub-routine for a quantum operator by augmenting the structural advantage (sparse and banded Toeplitz in our case) and the overall gate complexity in the quantum eigenvalue estimation.

In Fig. 6a, the quantum gate complexity versus the dimension (*N*) of the filter operator is given. The quantum simulation is performed for $t=0.2$ s, with the precision of the Hamiltonian approximation of $\varepsilon _P=0.01$, and the length of the ECG signal is given by $N=2351$. The quantum gates required by the proposed structured Hamiltonian simulation are much less than that of the standard quantum Hamiltonian simulation. For example, the quantum gate-operation complexity (with $N=1012$) of the standard QHS algorithm (considering the sparsity) is 731 approximately, whereas in the proposed QSF simulation (considering sparsity and banded-Toeplitz structure), the required cost is 374 approximately as shown in Fig. 6a. It reduces a gate cost of $48.84\%$ approximately for $N=1012$. Hence, the proposed QSF filter gives an advantage in terms of quantum resources compared to standard quantum methods for large ECG datasets.

In general, the matrix inversion for the filter operator ${\textbf{P}}\in {\mathbb {R}}^{N\times N}$ has a run-time complexity of ${\mathscr {O}}{(N^3)}$ and ${\mathscr {O}}(N^{2.37})$ approximately by Gauss-Jordan and Coppersmith-Winograd-based approaches^45^. The classical CG-based filters such as independent component analysis^46^ incur a run-time complexity of ${\mathscr {O}}\left( Nd\right)$. The EMD^44^ and DWT^1^ methods for ECG signal denoising have run-time of ${\mathscr {O}}(\beta _1 N \log N)$ with $\beta _1\in \tilde{{\mathscr {O}}}(1)$^47^, and $\tilde{{\mathscr {O}}(N)}$^48^ respectively. In Fig. 6b, we have shown a curve of run-time complexity for different filtering algorithms applied to ECG signal processing. In the simulation, we have kept the algorithmic error ($\varepsilon _P=0.01$) for both the classical and quantum set-up and kept the length of the signal vector to be $N=1024$. The Gauss-Jordan (denoted as *GJ*) and Coppersmith method (shown as *CW*) take significantly high run-time with data dimension. The classical algorithms- EMD, DWT (and CG) perform polynomial and linear time complexity approximately. The proposed quantum filter (denoted by $'QSF'$) initially showed similar performance to that of EMD and DWT. For large dimensional datasets ($N\ge 200$), the proposed QSF outperforms all classical algorithms. As an example, the run-time taken by EMD, DWT, and QSF is given by $1.008\times 10^6$, $10.1\times 10^{4}$, and $2.4\times 10^4$ respectively for $N=2^{10}$. Hence, with this experimental setup for banded Toeplitz and sparse filter matrices, the exponential speed-up can be augmented for large ECG datasets as reported.

### Simulation results on IBM quantum machine

**Figure 7:**
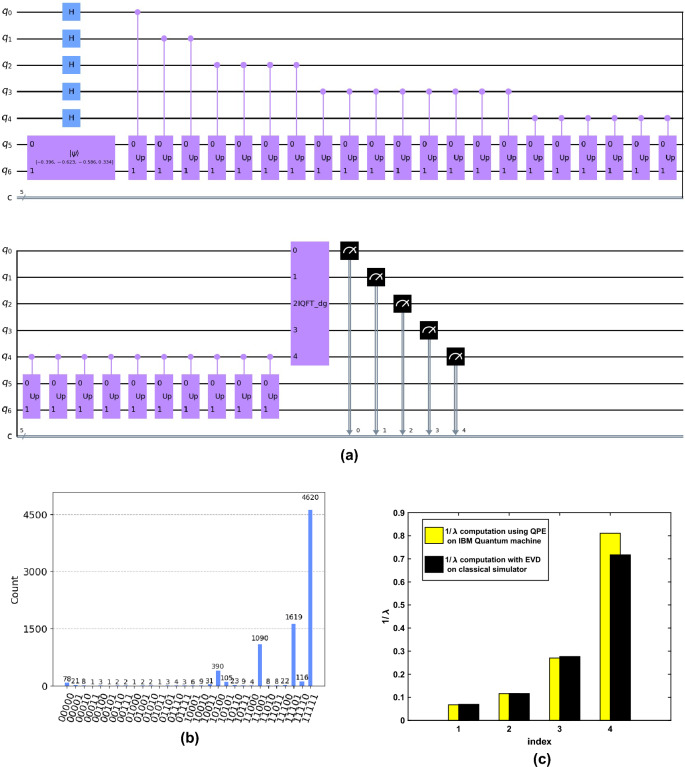
A quantum simulation of the proposed filter on IBM Quantum machine. (**a**) A QPE circuit designed on IBM Quantum machine. (**b**) Histogram for minimum eigenvalue estimation. (c) Comparison of estimated eigenvalues.

A small-scale quantum simulation of the proposed quantum filter is shown in Fig. 7 with the available quantum resources at the present moment. We consider a $4\times 4$ filter matrix ${\textbf{P}}$ and perform Hamiltonian simulation and QPE on the IBM quantum machine (‘statevector simulator’) with 5 qubit accuracy for the estimated eigenvalues. In Fig. 7a, a QPE circuit is shown for the minimum eigenvalue estimation, with five auxiliary qubits ($q_0,\dots , q_4$) and two qubits ($q_5, q_6$) for the given eigenstate (${|{\psi }\rangle }$) preparation. Here, $\text {'Up'}$ denotes the unitary operator corresponding to the filter matrix ${\textbf{P}}$ prepared through Hamiltonian simulation, and ’IQFT$\_$dg’ represents the inverse quantum Fourier transform sub-routine. The quantum measurement is performed on the computational bases (on the binary strings 00000 to 11111 based on the auxiliary qubits). A histogram is shown in Fig. 7b with 8192 quantum measurements for finding the minimum eigenvalue for the filter matrix ${\textbf{P}}$. From the histogram, the basis 11111 has shown the highest probability (with a count of 4620 out of 8192) with an estimated eigenvalue (here, it is the lowest eigenvalue of the filter) given by 1.394448. The proposed QSF filter performs the inverse of the diagonal matrix (with eigenvalues of the matrix ${\textbf{P}}$ in its diagonal). We have shown the reciprocal of the estimated eigenvalues (here it is $1/\lambda$) in Fig. 7c obtained using multiple QPE circuits simulated on an IBM quantum machine. As compared with eigenvalues obtained on a classical computer, the eigenvalues estimated on an IBM quantum machine are quite close.

### Analysis

The performance of the proposed algorithm is derived analytically in terms of mean square error (MSE) bound and computational complexity. The error analysis considers the quantum measurement error and truncation error in addition to classical observation noise. To show the computational resource efficiency, we propose a lemma on gate complexity and run-time complexity, as portrayed in this section.

#### Error analysis

##### Lemma 3

The mean square error of the proposed quantum filter is a function of the eigenvalues of the filter matrix, signal power, and noise power which can be given as a bound with the expression in (28), where $\sigma _x^2$ denotes the power of the information-bearing signal, and $\sigma _w^2={\mathbb {E}}\left( {\Vert {\textbf{w}} \Vert ^2}\right)$ and $\lambda _i$ is the $i^{th}$ eigenvalue of the filter matrix ${\textbf{P}}$.

28 $$\begin{aligned} err\le \sigma _x^2\left( 1- 2\sum _{i=1}^{N}{\mathbb {E}}\left( \frac{1}{\lambda _i} \right) + {\mathbb {E}} \sum _{i=1}^{N} \left( \frac{1}{\lambda _i^2}\right) \right) + {\mathbb {E}} \sum _{i=1}^{N} \left( \frac{1}{\lambda _i^2}\right) \sigma _w^2, \end{aligned}$$

##### Proof

Assuming the filter matrix ${\textbf{P}}^{-1}={\textbf{W}}$, the estimation error can be written as

29 $$\begin{aligned} err&=\Vert {\textbf{x}} -\hat{{\textbf{x}}}\Vert ^2 = {\mathbb {E}}\left( {\textbf{x}}-{\textbf{W}}{\textbf{y}}\right) ^{\dagger } \left( {\textbf{x}}-{\textbf{W}}{\textbf{y}}\right) \nonumber \\&={\mathbb {E}}\left( {\textbf{x}}^{\dagger }{\textbf{x}} \right) - {\mathbb {E}}\left( {\textbf{x}}^{\dagger }{\textbf{W}}{\textbf{y}} \right) - {\mathbb {E}}\left( {\textbf{y}}^{\dagger }{\textbf{W}}^{\dagger }{\textbf{x}} \right) + {\mathbb {E}}\left( {\textbf{y}}^{\dagger }{\textbf{W}}^{\dagger }{\textbf{W}}{\textbf{y}} \right) . \end{aligned}$$

Here, $\sigma _x^2={\mathbb {E}}\left( {\textbf{x}}^{\dagger }{\textbf{x}} \right)$ denotes the signal power. The second term in the R.H.S of expression (29) can be written as follows

30 $$\begin{aligned} e_2&={\mathbb {E}}({\textbf{x}}^{\dagger }{\textbf{W}}{\textbf{y}})\nonumber \\&= {\mathbb {E}}\left( {\textbf{x}}^{\dagger }{\textbf{W}}({\textbf{x}}+{\textbf{w}})\right) \nonumber \\&={\mathbb {E}}\left( {\textbf{x}}^{\dagger }{\textbf{W}}{\textbf{x}} \right) ~~~(\text {assuming }{\textbf{x}}\text { and }{\textbf{w}}\text { are independent})\nonumber \\&= {\mathbb {E}}\left( {\textbf{x}}^{\dagger }{\textbf{U}}^{\dagger }\Sigma ^{-1}{\textbf{U}}{\textbf{x}}\right) ~~~(\text {let,} {\textbf{P}}={\textbf{U}}\Sigma {\textbf{U}}^{\dagger })\nonumber \\&={\mathbb {E}} \left( {\textbf{v}}^{\dagger } \Sigma ^{-1}{\textbf{v}}\right) ~~~ (\text {let,} {\textbf{v}}={\textbf{U}}{\textbf{x}},\text { and }{\textbf{v}}=[v_1, v_2, \dots , v_N]^{\dagger }) \nonumber \\&={\mathbb {E}} \left( \sum _{i=1}^{N} \frac{1}{\lambda _i}\vert v_i \vert ^2 \right) ~\text {where, }\Sigma =\textbf{diag}(\lambda _1, \lambda _2, \dots , \lambda _N)\nonumber \\&\le \sum _{i=1}^{N} {\mathbb {E}}\left( \frac{1}{\lambda _i}\right) {\mathbb {E}}(\vert v_i \vert ^2)\nonumber \\&= \sigma _x^2 \sum _{i=1}^{N} {\mathbb {E}}\left( \frac{1}{\lambda _i}\right) ~~(\text {as }\Vert {\textbf{v}}\Vert _2=\Vert {\textbf{U}}{\textbf{x}}\Vert _2=\Vert {\textbf{x}}\Vert _2). \end{aligned}$$

Similarly, we obtain the following expression for the $3^{rd}$-term ${\mathbb {E}}\left( {\textbf{y}}^{\dagger }{\textbf{W}}^{\dagger }{\textbf{x}} \right)$ as

31 $$\begin{aligned} e_3={\mathbb {E}}\left( {\textbf{y}}^{\dagger }{\textbf{W}}^{\dagger }{\textbf{x}} \right) =\sigma _x^2 \sum _{i=1}^{N} {\mathbb {E}}\left( \frac{1}{\lambda _i}\right) . \end{aligned}$$

Note that, the expression of the fourth term ${\mathbb {E}}\left( {\textbf{y}}^{\dagger }{\textbf{W}}^{\dagger }{\textbf{W}}{\textbf{y}} \right)$ can be simplified as follows

32 $$\begin{aligned} e_4&= {\mathbb {E}}\left( {\textbf{y}}^{\dagger }{\textbf{W}}^{\dagger }{\textbf{W}}{\textbf{y}} \right) ={\mathbb {E}}\left( ({\textbf{x}}+{\textbf{w}})^{\dagger }{\textbf{W}}^{\dagger }{\textbf{W}}({\textbf{x}}+{\textbf{w}}) \right) \nonumber \\&= {\mathbb {E}}\left( {\textbf{x}}^{\dagger }{\textbf{W}}^{\dagger }{\textbf{W}}{\textbf{x}} \right) + {\mathbb {E}}\left( {\textbf{w}}^{\dagger }{\textbf{W}}^{\dagger }{\textbf{W}}{\textbf{w}} \right) \nonumber \\&={\mathbb {E}} \left( {\textbf{x}}^{\dagger } ({\textbf{U}}^{\dagger } \Sigma ^{-1} {\textbf{U}})^{\dagger } ({\textbf{U}} \Sigma ^{-1} {\textbf{U}}){\textbf{x}} \right) + {\mathbb {E}}\left( {\textbf{w}}^{\dagger }{\textbf{W}}^{\dagger }{\textbf{W}}{\textbf{w}} \right) \nonumber \\&= {\mathbb {E}} \left( {\textbf{x}}^{\dagger } {\textbf{U}}^{\dagger } \Sigma ^{-2} {\textbf{U}}{\textbf{x}} \right) + {\mathbb {E}}\left( {\textbf{w}}^{\dagger } {\textbf{U}}^{\dagger } \Sigma ^{-2} {\textbf{U}}{\textbf{w}} \right) \nonumber \\&= {\mathbb {E}} \left( {\textbf{v}}^{\dagger } \Sigma ^{-2} {\textbf{v}} \right) + {\mathbb {E}} \left( {\textbf{u}}^{\dagger } \Sigma ^{-2} {\textbf{u}} \right) ~\text {let,} {\textbf{u}}={\textbf{U}}{\textbf{w}}\nonumber \\&\le {\mathbb {E}}\sum _{i=1}^{N} \left( \frac{1}{\lambda _i^2}\right) {\mathbb {E}}\left( \Vert {\textbf{v}} \Vert ^2 + \Vert {\textbf{w}} \Vert ^2 \right) \nonumber \\&=(\sigma ^2_x +\sigma ^2_w){\mathbb {E}}\sum _{i=1}^{N} \left( \frac{1}{\lambda _i^2}\right) . \end{aligned}$$

Hence, the error is given by

33 $$\begin{aligned} err&= \sigma _x^2 - e_2 - e_3 +e_4 \nonumber \\&= \sigma _x^2 - 2 \sigma _x^2 \sum _{i=1}^{N} {\mathbb {E}} \left( \frac{1}{\lambda _i}\right) + \left( \sigma ^2_x +\sigma ^2_w\right) {\mathbb {E}}\sum _{i=1}^{N} \left( \frac{1}{\lambda _i^2}\right) \nonumber \\&= \sigma _x^2 \left( 1- 2{\mathbb {E}} \left( \frac{1}{\lambda _i}\right) + {\mathbb {E}}\sum _{i=1}^{N} \left( \frac{1}{\lambda _i^2}\right) \right) \nonumber \\&+\sigma ^2_w {\mathbb {E}}\sum _{i=1}^{N} \left( \frac{1}{\lambda _i^2}\right) . \end{aligned}$$

$$\square$$

Dropping the subscript *i* in $\lambda _i$, we can express its estimated value ${\hat{\lambda }}$ as

34 $$\begin{aligned} {\hat{\lambda }}= \lambda _{tr} + \lambda _{m} + \lambda _{\varepsilon } +\lambda _{w}, \end{aligned}$$

where $\lambda _{tr}$ denotes the true eigenvalue; $\lambda _{m}, ~\lambda _{\varepsilon }~$, and $\lambda _{w}$ represent the perturbation error in the estimated eigenvalue due to quantum measurement uncertainty, Hamiltonian simulation error and observation noise respectively. Hence, the total mean square error in the estimated eigenvalues has the following bound

35 $$\begin{aligned} \Vert {\hat{\lambda }} - \lambda _{tr} \Vert ^2 \le \vert \varepsilon _{P}\vert ^2 +\sigma _m^2 + \sigma _w^2, \end{aligned}$$

where $\vert \varepsilon _P\vert ^2$ denotes the Hamiltonian simulation error, $\sigma _m^2$ is the variance of the measurement uncertainty, and the $\sigma _w^2$ is the variance of the observation noise. With a precision of $N_q$ qubits for the estimated eigenvalues, the probability of error due to measurement uncertainty is given by

36 $$\begin{aligned} p(\lambda _m = k)&= \sum _{k=1}^{2^{N_q}-1} p(\lambda _m|{\hat{\lambda }})p({\hat{\lambda }})\nonumber \\&= \frac{1}{2^{N_q}}{^lC_k} {P_b^{l -k} \left( 1-P_b \right) ^k}~\text {with}~l=2^{N_q}, \end{aligned}$$

where *k* denotes an integer representation of the parameter $\lambda _m$ in a binary string, and this represents the number of errors in the string. The variance of the measurement noise parameter $\lambda _m$ for a $N_q$-bit resolution can be expressed following^49^ as (37).

37 $$\begin{aligned} \sigma _m^2&= \frac{1}{2^{N_q} \sum _{l=1}^{N_q}{}^{N_q}C_{j}\times N_q} \sum _{i=1}^{2^{N_q}} \sum _{j=1}^{N_q} \sum _{k=1}^{{}^{N_q}C_{j}} p_b^j(1-p_b)^{N_q -j}\Vert d_i - d_{i,j}^k \Vert ^2,~\text {with}~p_b \nonumber \\&= \frac{1}{N}\sum _{k=1}^{N}\sum _{j} B_j \prod _{r=1}^{R_n} \cos ^2 \left( \frac{\tau _r{\hat{\lambda }}_i}{2} + \frac{\beta _r - m_r \pi }{2} \right) . \end{aligned}$$

Here, $d_i$ is the decimal value of the $i^{th}$ binary-string representation (of length $N_q$) of an eigenvalue, $d_{i,j}^k$ denotes an $N_q$-length binary string with *j* bits reversed with respect to $d_i$ and *k* represents the $k^{th}$ realization of the string.

The probability of a bit’s (in the binary representation of an eigenvalue) correct measurement outcome is given by

38 $$\begin{aligned} p_b = \displaystyle \frac{1}{N}\sum _{k=1}^{N}\sum _{j} B_j \prod _{r=1}^{R_n} \cos ^2 \left( \frac{\tau _r{\hat{\lambda }}_i}{2} + \frac{\beta _r - m_r \pi }{2} \right) , \end{aligned}$$

where ${\hat{\lambda }}_i$ is the *i* *th* estimated eigenvalue, $\tau _r$-times unitary rotation in each quantum measurement, $m_r\in \{0,1\}$, $B_j$ denotes normalizing coefficients, and $\beta$ represents the the phase for rotation around the *Z*-axis. The terms ${\mathbb {E}}\left( \frac{1}{{\hat{\lambda }}}\right)$, and ${\mathbb {E}}\left( \frac{1}{{\hat{\lambda }}^2}\right)$ can be expressed (assuming independence of $\lambda _{tr},\lambda _m, \lambda _{\varepsilon }$, and $\lambda _w$) as follows

39 $$\begin{aligned} {\mathbb {E}}\left( \frac{1}{{\hat{\lambda }}}\right)&= {\mathbb {E}}_{\lambda _m} \left( \frac{1}{\sigma _w\sqrt{2\pi }}\int _{0}^{\infty } \frac{1}{K+ \lambda _w} \exp {\left( \frac{-\lambda _w^2}{2\sigma _w^2} \right) } d\lambda _w \right) ~ \nonumber \\ {\mathbb {E}}\left( \frac{1}{{\hat{\lambda }}^2}\right)&= {\mathbb {E}}_{\lambda _m} \left( \frac{1}{\sigma _w\sqrt{2\pi }}\int _{0}^{\infty } \frac{1}{(K+ \lambda _w)^2} \exp {\left( \frac{-\lambda _w^2}{2\sigma _w^2} \right) } d\lambda _w \right) ~ \nonumber \\&\text {where}~ K=\lambda _{tr} +\lambda _{m}+\lambda _{\varepsilon }. \end{aligned}$$

The integrals $I_1=\int \frac{1}{K+ \lambda _w} \exp {\left( \frac{-\lambda _w^2}{2\sigma _w^2} \right) } d\lambda _w$ and $I_2=\int _{0}^{\infty } \frac{1}{(K+ \lambda _w)^2} \exp {\left( \frac{-\lambda _w^2}{2\sigma _w^2} \right) } d\lambda _w$ are mathematically intractable and divergent for the limit $\left[ 0,\infty \right)$. We take the truncated Taylor series about the point $\lambda _w=0$ and step size *h* for the exponential term as

40 $$\begin{aligned} \exp {\left( \frac{-\lambda _w^2}{2\sigma _w^2} \right) } = \sum _{l=0}^{\infty } \left( \frac{-\lambda _w^2}{2\sigma _w^2}\right) = 1 - \frac{\lambda _w^2}{2\sigma _w^2} + {\mathscr {O}}(h^2), \end{aligned}$$

where ${\mathscr {O}}(h^2)$ is the residual error. Further, as the integral is divergent in $\left[ 0,\infty \right)$, we fix an upper bound for $\lambda _w$ as $u_{max}$ which is often the case in practical systems. Hence, the integral $I_1$ can be solved for $\lambda _w$ as follows

41 $$\begin{aligned} I_1&=\lim _{u \rightarrow \infty } \int _{0}^{u} \frac{1}{K+ \lambda _w} \exp {\left( \frac{-\lambda _w^2}{2\sigma _w^2} \right) } d\lambda _w\nonumber \\&\le \lim _{u \rightarrow u_{max}} \int _{0}^{u} \frac{1}{K+ \lambda _w} \left[ 1 - \frac{\lambda _w^2}{2\sigma _w^2}\right] d\lambda _w~\nonumber \\&\approx \frac{-1}{2\sigma _w^2}\int _{0}^{u_{max}} \frac{\lambda _w^2 - 2\sigma _w^2}{K+\lambda _w} d\lambda _w=\frac{-1}{2\sigma _w^2}\left[ I_{11}\right] _{u=0}^{u=u_{max}}, \end{aligned}$$

where,

42 $$\begin{aligned} I_{11}&= \int \frac{\lambda _w^2 - 2\sigma _w^2}{K+\lambda _w} d\lambda _w \nonumber \\&\hbox { Let,}\ z=\lambda _w+K,~dz=d\lambda _w\nonumber \\&=\int \frac{(z-K)^2 - 2\sigma _w^2}{z}dz\nonumber \\&=\frac{z^2}{2} + (K^2 - 2\sigma _w^2) \ln {z} - 2Kz +C_1, \nonumber \\&C_1\text { be arbitrary constant}\nonumber \\&=(K^2 - 2\sigma _w^2)\ln \vert \lambda _w +K \vert + \frac{(\lambda _w + K)^2}{2} - 2K(\lambda _w +K)+C_1 \end{aligned}$$

Hence, the integral $I_1$ becomes

43 $$\begin{aligned} I_1&\le -\frac{(K^2 - 2\sigma _w^2)\ln \vert \lambda _w +K\vert }{2\sigma _w^2} -\frac{(\lambda _w+K)^2}{4\sigma _w^2} + \frac{K(\lambda _w + K)}{\sigma _w^2} +C_2,\nonumber \\&= \frac{(4\sigma _w^2-2K^2)\ln \vert \lambda _w + K\vert - \lambda _w^2 + 2K\lambda _w}{4\sigma _w^2} +C_3. \end{aligned}$$

Here, $C_2={-2\sigma _w^2}C_1$, and $C_3=4\sigma _w^2 C_2$. Considering, $u_{max}>0$, and $u_{max}+K>0$, the expression of $I_1$ can be simplified with the definite integral limit in $\left[ 0,u_{max}\right]$ as given by (47). Similarly, considering Taylor series approximation for the exponential part (with two terms), and solving $I_2$ with upper limit $u_{max}$ we obtain

44 $$\begin{aligned} I_2&\le \lim _{u\rightarrow \infty } \int _{0}^{u} \frac{1}{(K+ \lambda _w)^2} \left( 1 - \frac{\lambda _w^2}{2\sigma _w^2} \right) d\lambda _w\nonumber \\&\approx \frac{-1}{2\sigma _w^2} \int _{0}^{u_{max}} \frac{\lambda _w^2 - 2\sigma _w^2}{(K+\lambda _w)^2} d\lambda _w = \frac{-1}{2\sigma _w^2} \left[ I_{22}\right] _{u=0}^{u=u_{max}}, \end{aligned}$$

where,

45 $$\begin{aligned} I_{22}&= \int \frac{\lambda _w^2 - 2\sigma _w^2}{(K+\lambda _w)^2} d\lambda _w\nonumber \\&\hbox {let,} z=\lambda _w+K \hbox {, and} dz=dx\nonumber \\&= \int \frac{(z-K)^2 - 2\sigma _w^2}{z^2} dz,\nonumber \\&= -2K \int \frac{1}{z} dz + (K^2 -2\sigma _w^2)\int \frac{1}{z^2}dz + \int dz \nonumber \\&= -2K \ln {\vert \lambda _w +K\vert } + (\lambda _w + K) -\frac{K^2 - 2\sigma _w^2}{\lambda _w + K} + C_4, \end{aligned}$$

where $C_4$ is an arbitrary constant. Hence the integral $I_2$ becomes

46 $$\begin{aligned} I_2&\le \frac{K\ln {\vert \lambda _w +K \vert }}{\sigma _w^2} + \frac{K^2 - 2\sigma _w^2}{2\sigma _w^2(\lambda _w +K)} - \frac{(\lambda _w+ K)}{2\sigma _w^2} + C_5, \end{aligned}$$

where $C_5=-2\sigma _w^2 \times C_4$. Considering, $u_{max}>0$, and $u_{max}+K>0$, the expression of $I_2$ can be simplified with the definite integral limit in $\left[ 0,u_{max}\right]$ as given by (48). Taking the bounds for the integrals $I_1$ in (47) and $I_2$ in (48), and considering the probability mass function in (36) the expressions ${\mathbb {E}}(\frac{1}{{\hat{\lambda }}})$, and ${\mathbb {E}}(\frac{1}{{\hat{\lambda }}^2})$ for *M* quantum measurements can be approximated by (49) and (50) respectively.

47 $$\begin{aligned}{}&I_1 \le \frac{(4\sigma _w^2 -2K^2)\ln (u_{max}+K)-u_{max}^2 +2Ku_{max}}{4\sigma _w^2} + \frac{K^2 \ln K}{2\sigma _w^2} - \ln {K}, ~\text {with}~K=\lambda _{tr} +\lambda _{m}+\lambda _{\varepsilon }. \end{aligned}$$

48 $$\begin{aligned}&I_2\le \dfrac{\left( 2Ku_{max}+2K^2\right) \ln \left( u_{max}+K\right) -u_{max}^2-Ku_{max}-2{\sigma }_\text {w}^2+K^2}{2{\sigma }_\text {w}^2u_{max}+2K{\sigma }_\text {w}^2}+\dfrac{2{\sigma }_\text {w}^2-2K^2\ln \left( K\right) -K^2}{2K{\sigma }_\text {w}^2} ~\text {for}~u_{max}>0, K>0. \end{aligned}$$

49 $$\begin{aligned}&{\mathbb {E}}\left( \frac{1}{{\hat{\lambda }}}\right) \approx \frac{1}{\sigma _w\sqrt{2\pi }}\sum _{i=1}^{M} \left( \log ({\lambda _{tr} +\lambda _{m}(i)+\lambda _{\varepsilon }}+ u_{max})\left[ 1 + \frac{({\lambda _{tr} +\lambda _{m}(i)+\lambda _{\varepsilon })}^2}{2\sigma _w^2} \right] \right) p(\lambda _m (i))\nonumber \\&- \sum _{i=1}^{M} \left( \log (\lambda _{tr} +\lambda _{m}(i)+\lambda _{\varepsilon }) \left[ 1 -\frac{({\lambda _{tr} +\lambda _{m}(i)+\lambda _{\varepsilon })}^2}{2\sigma _w^2}\right] + \frac{u_{max}^2 - 2u_{max}({\lambda _{tr} +\lambda _{m} (i)+\lambda _{\varepsilon }})}{4\sigma _w^2} \right) p(\lambda _m (i)). \end{aligned}$$

50 $$\begin{aligned}&{\mathbb {E}}\left( \frac{1}{{\hat{\lambda }}^2}\right) \approx \frac{1}{\sigma _w\sqrt{2\pi }}\sum _{i=1}^{M} \left( \dfrac{\left( 2Ku_{max}+2K^2\right) \ln \left( u_{max}+K\right) -u_{max}^2-Ku_{max}-2{\sigma }_\text {w}^2+K^2}{2{\sigma }_\text {w}^2u_{max}+2K{\sigma }_\text {w}^2} +\dfrac{2{\sigma }_\text {w}^2-2K^2\ln \left( K\right) -K^2}{2K{\sigma }_\text {w}^2} \right) p(\lambda _m (i)),\nonumber \\&~\text {with}~ K=\lambda _{tr} +\lambda _{m}(i)+\lambda _{\varepsilon }, ~\text {and }M\text { measurements}. \end{aligned}$$

The quantum simulation-based quantum filter introduces additional perturbation due to the Hamiltonian approximation error $\varepsilon _p$ and the quantum measurement uncertainty and Hardware imperfection. For the large dimensional sample size, we assume that the total simulation uncertainty is white and zero-mean Gaussian distributed with variance given by $\sigma _e^2= \vert \varepsilon _p \vert ^2 + \sigma _m^2 +\sigma _h^2$, where $\sigma _m^2$ denotes the measurement uncertainty, and $\sigma ^2_h$ is the variance of hardware imperfection error (often occurs in qubit preparation and thermal instability). The effective quantum filter operator can be written as

51 $$\begin{aligned} \hat{{\textbf{P}}}&={\textbf{I}} + \eta {\textbf{D}}^T{\textbf{D}} + {\textbf{U}}\Sigma _p{\textbf{U}}^{\dagger }\nonumber \\&= {\textbf{U}} \left[ {\textbf{I}} + \eta ({\textbf{D}}{{\textbf{U}}})^{\dagger } ({\textbf{D}}{\textbf{U}}) +\Sigma _e \right] {\textbf{U}}^{\dagger }, \end{aligned}$$

where $\Sigma _e$ is the diagonal matrix corresponding to the error covariance matrix (due to simulation uncertainty). Hence, the estimated signal as shown in (4) is impacted by the inverse of the matrix $\hat{{\textbf{P}}}$ given by

52 $$\begin{aligned} \hat{{\textbf{x}}}&=(\hat{{\textbf{P}}})^{-1}{\textbf{y}}\nonumber \\&= \left( {\textbf{P}} + {\textbf{P}}_e \right) ^{-1}{\textbf{y}},\nonumber \\&= \left( {\textbf{U}}{\Sigma }{\textbf{U}}^{\dagger } + {\textbf{U}}{\Sigma }_e{\textbf{U}}^{\dagger }\right) ^{-1} {\textbf{y}} \nonumber \\&= {\textbf{U}}^{\dagger } \left( \Sigma + \Sigma _e\right) ^{-1} {\textbf{U}}{\textbf{y}} ~~(\text {as}, {\textbf{U}}^{\dagger }={\textbf{U}}). \end{aligned}$$

Note that the iterative execution of the proposed algorithm perceives the filter perturbation in eigenvalues with the variance of $\sigma _e^2$. Hence, multiple quantum measurements are required to find the correct basis from the histogram and to get the estimated ECG signal $\hat{{\textbf{x}}}$. The estimated error in $1/\lambda$ perceived in the quantum simulator is shown in Fig.7c.

In the proposed QSF, we have exploited both the sparsity and banded-Toeplitz structure of the Hamiltonian matrix ${\textbf{P}}$. First, the quantum gate-complexity advantage is discussed in comparison with the standard QHS method. Secondly, we analyse the quantum run-time complexity of the proposed QSF method in comparison with standard classical filtering methods for ECG signals.

#### Complexity of the Hamiltonian simulation

The quantum gate-operation complexity for the QHS with a sparse Hamiltonian matrix ${\textbf{P}}\in {\mathbb {C}}^{N\times N}$ in time *t* and approximation error $\varepsilon _P$ is given in^35,50^ as

53 $$\begin{aligned} {\mathscr {O}}{\left( \frac{n_q\log ^2(\frac{T}{{\tilde{\varepsilon }}_P})}{\log \log (\frac{T}{{\tilde{\varepsilon }}_P})} \right) }. \end{aligned}$$

Here $n_q=\log (N)$ denotes the input size of qubit for the *d*-sparse matrix ${\textbf{P}}$, and *T* is defined as $T=d^2\Vert {\textbf{P}} \Vert _{\max } t$.

In this work, we have augmented the structural advantage of the Banded-Toeplitz matrix pattern. The proposed Algorithm 1 has shown a sparse decomposition of the matrix ${\textbf{P}} \in {\mathbb {C}}^{N\times N}$ with $5N-6$ non-zero elements (instead of operations for $N^2$) and the sparsity $d=5$. In the below proposition, we show the computational complexity of the proposed structured QHS.

##### Lemma 4

The computational gate-operation complexity for simulating a Banded-Toeplitz structured operator ${\textbf{P}}\in {\mathbb {C}}^{N\times N}$ using Algorithm 1 to prepare an approximate unitary ${\textbf{U}}_P\in {\mathbb {C}}^{N\times N}$ within the evolution time *t* and precision $\varepsilon _P$ is given by

54 $$\begin{aligned} \tilde{{\mathscr {O}}}\left( \sqrt{\beta }\log {N}\frac{\log ^2 \left( \beta \Vert {\textbf{P}}\Vert _{max} \frac{t}{\varepsilon _P}\right) }{\log \log \left( \beta \Vert {\textbf{P}}\Vert _{max} \frac{t}{\varepsilon _P}\right) } \right) ~\text {with} ~\beta =\tilde{{\mathscr {O}}}(1). \end{aligned}$$

##### Proof

The banded-Toeplitz matrix with band *d* has a classical inversion cost of $\tilde{{\mathscr {O}}}(d\log {N})$^51^. Here, the filter matrix with Kernel $[1~-2~1]$ has the band length $d=5$ with the Toeplitz structure. Encoding such an operator for $5N-6$ matrix elements with band $d=5$ will incur a gate cost approximately $\tilde{{\mathscr {O}}}\left( {d\log N}\right)$ following (53) with $n_q=\log {N}$, and $T\approx 25 \Vert {\textbf{P}}\Vert _{max} t$. Hence, the overall gate complexity of the Hamiltonian simulation becomes

$$\begin{aligned} \tilde{{\mathscr {O}}}\left( \sqrt{\beta }\log {N} \frac{\log ^2 \left( \beta \Vert {\textbf{P}}\Vert _{max} \frac{t}{\varepsilon _P}\right) }{\log \log \left( \beta \Vert {\textbf{P}}\Vert _{max} \frac{t}{\varepsilon _P}\right) } \right) ~\text {with} ~\beta =\tilde{{\mathscr {O}}}(1). \end{aligned}$$

$$\square$$

#### Complexity of the quantum filter

In filtering large-dimensional biomedical signals such as ECG, one critical aspect is the filter’s complexity. To put our quantum filter in context, we compare it with the classical filtering approaches. Here, the filter is designed as the inversion of the matrix ${\textbf{P}} \in {\mathbb {R}}^{N\times N}$ whereby regularizing with a factor $\lambda$ which improves condition number $\kappa$ of ${\textbf{P}}$ in the presence of perturbation. In addition to the sparsity *d*, the underlying filter has a Toeplitz structure, which gives the computational gate complexity advantage, as discussed earlier.

In the implementation of the filer, most of the classical algorithms for the matrix inversion, such as the Gauss-Jordan method, take a run time of ${\mathscr {O}}(N^3)$. Some optimized classical algorithms such as the Coppersmith-Winograd-based method and its variants take ${\mathscr {O}}(N^{2.3 + \gamma })$ with the constant $\gamma >0$^45^. One of the best classical matrix inversion approaches is the conjugate gradient algorithm which incurs a total run-time of ${\mathscr {O}}\left( Nd\sqrt{\kappa }\log {(\frac{1}{\varepsilon })}\right)$ considering the operator ${\textbf{P}}$ be positive definite, and the precision is given by $\varepsilon _P$.

##### Lemma 5

There is an efficient algorithm for simulating the filter matrix ${\textbf{P}}^{-1}$ having a regularized condition number $\kappa$, a constant sparsity (*d*), and large dimension (*N*) with quantum run time complexity given by

55 $$\begin{aligned} \tilde{{\mathscr {O}}}\left( \frac{\kappa ^2 \text {poly}(\log {N})}{\varepsilon _P}\right) . \end{aligned}$$

##### Proof

Following HHL quantum matrix inversion^27^, the run-time complexity for the sparse-matrix (${\textbf{P}}$) inversion can be obtained as $\tilde{{\mathscr {O}}}\left( \frac{d^2 \kappa ^2 \log (N)}{\varepsilon _P}\right)$. For *N* times execution, the total run-time of ${\mathscr {O}}(cN\log {N})$ with $c=d^2 \kappa ^2$. Employing the strategies as shown in^52^, this can be further improved to $\tilde{{\mathscr {O}}}\left( \frac{c \log (N^2)}{\varepsilon _P}\right)$. Here, $c=25\kappa ^2$ for the penta-diagonal structure of the filter matrix (with $d=5$). Hence, the overall time complexity of the proposed Quantum filter is approximately $\tilde{{\mathscr {O}}}\left( \frac{\kappa ^2 {poly}(\log {N})}{\varepsilon _P}\right)$. $\square$

## Discussion

### Note on $\lambda$ trade-off for sharp change and smoothing in different time segments of the denoised ECG signal

 One of the critical aspects of a filter applied to the ECG signal is that the $'QRS'$ complex is preserved with proper detection of the $'R'$ and $'S'$ patterns while the $'PQ'$ and $'ST'$ segments do not perturb much after denoising. In the QRS complex, the time domain transition (or gradient) of the signal is sharp, whereas, in the $'PQ'$ and $'ST'$, it is slowly growing and flat (or linear), respectively. Hence, a filter with high detectability of sharp edges may often perturb the $'PQ'$ and $'ST'$ regions in the reconstructed signal due to large weights of the higher-order derivative terms present in the filter. On the other side, one may expect smoothness in the $'PQ'$ and $'ST'$ segments at the cost of reduced energy levels in the $'QRS'$ complex. In our proposed algorithm, the distribution of the weights to the derivative terms in the filter given in (2) is controlled by the parameter $\lambda$ of the filter operator ${\textbf{P}}$. One may find an optimization over $\lambda$ to find the minimum global error of the denoised ECG signal. However, the choice of $\lambda$ can be variably optimum with specific regions of the ECG. Note that the $'QRS'$ complex is related to ventricular depolarization, the $'PQ'$ interval is related to the electrical activity of the movement between the atria and the ventricles, and the $'ST'$ has a correlation with ventricular contraction (Chapter 1.3 in^53^). In our experimental simulation, we have seen that a lower value of $\lambda$ (typically $\frac{N}{25}$) gives a closer pattern for the $'QRS'$ complex, and a relatively higher value of $\lambda$ (approximately $1.2\times N$) gives a smooth pattern for $'PQ'$ and $'ST'$ segments respectively for a *N*-length ECG signal. Each segment and peak has its own importance relative to the health of the heart. For example, pre-excitation syndromes may occur for a shortened $'PQ'$ segment, and pericarditis and pulmonary embolism may show $'ST'$-segment abnormality. The *QRS* complex has multiple sharp transitions within a shorter span (less than 0.12 s). Abnormality in the $'QRS'$ complex may incur bundle-branch block, pre-excitation syndromes, and premature ventricular contraction etc. Here, we show the two different choices of $\lambda$, which preserve the pattern of both sharp and flat patterns in the denoised ECG signal through our proposed QSF.

### Note on accuracy and complexity trade-off

 In this article, a conceptually novel quantum filtering framework is proposed. The main motivation has been lying within the augmentation of quantum computational speed-up in the signal denoising application, focusing on the ECG signal. There are several future scopes to improvise the algorithm in order to increase the accuracy of the signal reconstruction from its noisy version. One can approach methods such as band-stop smoothing filter (BSSF) as shown in^25^ to get better filter response for ECG signal denoising. However, the complexity advantage due to the sparsity nature of the filter can be compromised with BSSF within quantum formalism. With the increase in the number of terms of Taylor series truncation for QHS, one can hope for a slight improvement in the accuracy at the cost of increased computational complexity. An increasing number of qubits (especially the ancillary qubits considered for representing the information) can provide us with improvement in the precision of the estimated signals. Hence, we see that the proposed QSF has a trade-off between complexity and accuracy. In this work, computational gate complexity and run-time complexity are exploited without compromising the accuracy of the filter as compared to the classical methods.

### Note on condition number ($\kappa$), and time of evolution (t)

 Two important parameters of the proposed QSF are the condition number ($\kappa$) and quantum time evolution (*t*). The QHS method requires a minimum time *t* for its optimal simulation. Note that we have chosen $t=0.2$ second using the concept of quantum time resolution as given in^49^. In general, the condition number for the matrix ${\textbf{P}}$ should be sufficiently less in order to maintain the positive definite property of the operator and its stable inversion. However, the perturbation from multiple sources (such as noise and quantum uncertainty phenomena) may increase the value of $\kappa$ and direct inversion with HHL sub-routine^27^ may degrade the filter performance. However, the regularization within the filter matrix (with tuning parameter $\lambda$) takes care of the matrix perturbation, and the condition number remains within a lower range. Hence, the proposed quantum filter may be applied in the perturbed situation and inherent regularization stabilizes the filter performance.

### Note on possible extension to quantum machine learning algorithms

 The recent deep learning (DL) models^54–56^ show potential avenues for the novel ECG signal denoising problem, surpassing the accuracy limits of the conventional statistical signal processing-based algorithms. However, the successful application of the DL largely depends on the training data dimensionality, which incurs significant computational complexity. In addition, training DL models require large computational resources (number of quantum registers and circuit depth in terms of quantum formalism), limiting their deployment in near-term quantum computers. Our work shows a quantum formalism for the quantum smoothing filter, with its possible integration in the NISQ computing and resource-limited quantum computers. Further, it remains an open research question of how quantum DL models, such as quantum convolutional neural networks^57^ can be utilized, enabling the proposed quantum filter to achieve high-precision quantum denoising and classification with the increasing capability of superconducting qubit technology in the biomedical domain.

### Note on the application of QSF in quantum communication and quantum internet

Quantum noise has been a bottleneck for problems in NISQ computing, such as quantum communications and quantum internet^58^. Within the realm of quantum internet^58^, there’s remarkable scope for quantum architecture, including utilising both unentangled and entangled structures. Additionally, scalable models for distributed gate-model quantum computation in near-term quantum systems have been put forward^59^. Literature suggests that valuable information in the context of the quantum internet often exhibits noisy characteristics, with one of the primary sources of error being the circuit complexity at the gate level. To address this, the proposed quantum smoothing filter could be beneficial, particularly for reducing gate complexity and noise in the quantum internet setting.

## Conclusion

A quantum smoothing filter is proposed for denoising information-bearing signals corrupted by observation noise. The proposed quantum algorithm exploits the penta-diagonal banded-Toeplitz matrix structure for sparse decomposition of the Hamiltonian matrix, which augments the quantum gate-complexity advantage compared to the standard Hamiltonian simulation. Compared with classical filtering techniques such as DWT and EMD, the quantum filter shows an advantage in run-time complexity. A study is performed on ECG signal denoising, and the performance analysis is given with accuracy and complexity for the proposed quantum filter framework. The results reported in this article show potential applications for signal filtering with large dimensions, such as ECG, using near-term quantum computers.

## Data Availability

The datasets supporting the current study are taken from the publicly available PhysioNet database^43^.

## Code availability

The code developed in this study is available upon request from the corresponding author.
